# The impact of the COVID-19 pandemic on the quality of life of head and neck cancer survivors

**DOI:** 10.1007/s00520-021-06198-6

**Published:** 2021-04-15

**Authors:** Oreste Gallo, Chiara Bruno, Luca Giovanni Locatello, Federica Martelli, Maria Cilona, Pietro Orlando, Giuseppe Fancello, Giandomenico Maggiore, Francesca Viberti, Pierguido Ciabatti, Simone Boccuzzi, Marco Mandalà

**Affiliations:** 1 Department of Otorhinolaryngology, Careggi University Hospital, Largo Brambilla 3, 50134 Florence, Italy; 2 Department of Otorhinolaryngology, Ospedale San Donato, Arezzo, Italy; 3 Department of Otorhinolaryngology, Ospedale della Misericordia, Grosseto, Italy; 4 Department of Otorhinolaryngology, Azienda Ospedaliera Universitaria Senese, Siena, Italy

**Keywords:** COVID-19, Head and neck cancer, Quality of life, Total laryngectomy, Lockdown

## Abstract

**Background:**

Head and neck cancer (HNC) survivors are particularly vulnerable to the deleterious consequences of lockdown and social distancing. The psychosocial effects of the COVID-19 pandemic on this group are still unknown, and we want to explore how their quality of life (QoL) has changed in this unique situation.

**Materials and methods:**

An online survey, composed of pandemic-specific items, plus the EORTC QLQ-C30, was administered to a cohort of HNC survivors. Using previously published reference values as a control group, we have evaluated the impact of the pandemic on their QoL. We have also explored the differences between those who had received a total laryngectomy (LP, laryngectomized population) vs other HNC patients, in order to assess the role of tracheostomy in this regard.

**Results:**

One hundred and twenty-one HNC patients completed the survey. The scores of the physical (80.5 vs 85, *p* = 0.028), role (78 vs 84, *p* = 0.030), and emotional functioning (76 vs 81, *p* = 0.041) were significantly different in the two groups, with worse functioning in our patients. Comparing LP with the other HNC patients, social (76.6 vs 88.9, *p* = 0.008) and physical functioning (75.5 vs 86.1, *p* = 0.006) were significantly worse in the former group. LP also reported a greater perception that others are afraid to be close to them (1.67 vs 1.32, *p* = 0.020). No differences were found between LP with and without voice prosthesis.

**Conclusions:**

Our results show how HNC patients are at high risk for a worsening in QoL because of the ongoing COVID-19 global pandemic.

## Introduction

COVID-19 has deeply changed our lives as over 116 million people have been infected worldwide [1]. The field of oncology has not been spared from such an unprecedented pandemic, and almost one out of three cancer patients were negatively affected in terms of both treatment and clinical care [2]. Furthermore, the pandemic has dramatically modified how hospital and outpatient care is delivered; in addition, access to many medical and surgical services has been restricted or converted into telemedicine consultations [3].

Head and neck cancer (HNC) patients are one of the most vulnerable groups in this respect: the majority being elderly, the presence of an immunocompromised state (both cancer- and treatment-related), and a long smoking history all are common features of both HNC and severe COVID-19 [4]. Moreover, through the temporary or permanent tracheostomy, airborne viral particles can immediately reach the lower airways and, in case of infection, these subjects might spread SARS-CoV-2 infection more efficiently, because of their altered anatomy and of the aerosolization of tracheal secretions [5–8]. HNC survivors generally report a low quality of life (QoL) compared to the general population and they are at double risk to commit suicide compared to other cancers [9, 10]. During the lockdown period, the majority of these patients were forced at home, torn between the fear of in-hospital exposure and the anxiety because of missed or delayed follow-up visits. To date, only very few publications have investigated the impact of the COVID-19 pandemic on the QoL in the cancer population and none is specific for HNC [11, 12]. This study was undertaken to explore more objectively the direct and indirect psychosocial effects of the COVID-19 pandemic on the QoL and well-being of a specific HNC survivor cohort.

## Materials and methods

### Selection of patients and definition of outcomes

In this cross-sectional and multi-institutional study, after IRB approval (CEAVC Reference Number 17961), from the 15th of March to the 15th of May, we asked patients to complete an anonymous online survey (Google Form®, Mountain View, CA, USA) during the telemedicine visits made by the Departments of Otorhinolaryngology in four Tuscan hospitals (Careggi University Hospital, Florence; Le Scotte University Hospital, Siena; San Donato Hospital, Arezzo; Misericordia Hospital, Grosseto). We included all HNC patients previously treated by surgery, chemoradiotherapy, or a combination of both, and with no evidence of disease at least in the past year. Exclusion criteria were the unwillingness to take part in the study, patients affected by known cognitive disorders, active HNC or suspected cancer recurrence, patients with a recent/past diagnosis of SARS-CoV-2 infection, or who have been living with people affected by SARS-CoV-2. We decided to exclude the latter, as the disease itself and the fear of possible complications for oneself and loved ones would have influenced the results of the questionnaire.

The survey consisted of several general questions to assess the participants’ demographics and clinical history, the cancer-specific Quality of Life Core Questionnaire (QLQ-C30) of the European Organization for Research and Treatment of Cancer (EORTC) version 3, and an expressly developed COVID-19 HNC Respiratory Questionnaire [13]. Data about sex, age, smoking history (yes/no plus the pack-years, when available), the anatomical site of HNC, the type of and time elapsed from previous treatment for HNC, and the presence of other respiratory conditions (COPD and asthma) were collected. The EORTC QLQ-C30 was used to assess the QoL: this is subdivided into scales (functional or symptom), composed of one to five items, all measured by a 4-point Likert-like method. Global QoL is instead scored on a 7-point Likert-like system. For each scale, a summary score was calculated according to the EORTC manual [14]. A higher score represents a better outcome on each domain for functional scales (i.e., QoL, physical, role, cognitive, social, and emotional functioning), and a worse outcome for symptom scales (i.e., dyspnea, insomnia, and financial difficulties). We used only the core module EORTC QLQ-C30 and not the HN35/HN43 because the latter is heavily treatment-dependent, and thus, it is supposed not to be directly influenced by the current pandemic [14]. The COVID-19 HNC Respiratory Questionnaire is presented in Table 1: it consisted of seven questions about perceived changes in one’s own and others’ behaviors and in quality of the air, measured by a 4-point Likert-like scale; finally, one multiple choice question was reserved for laryngectomized patients about the use of facemasks. This tool was devised only for informative purposes, given no validated or specific questionnaire exists, to the best of our knowledge, to assess how HNC patients perceive the current situation (mass masking, lockdown measures, etc.).

**Table 1:**
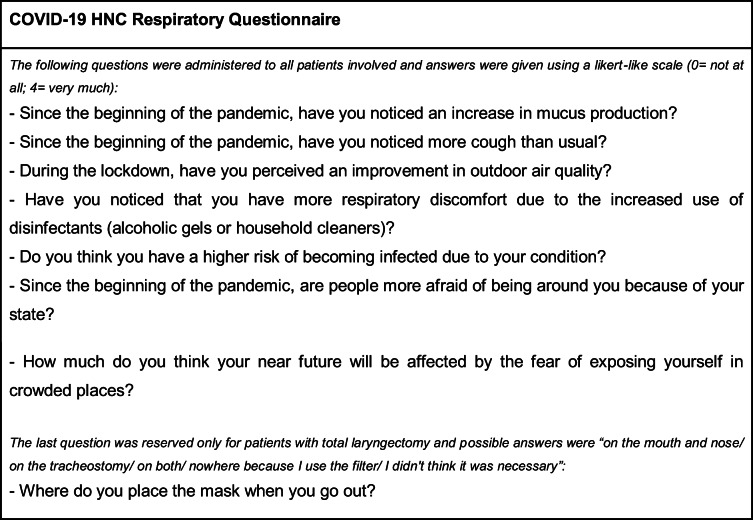
The COVID-19 HNC Respiratory Questionnaire

### Selection of the historical controls and comparison of scores

Because of the ongoing pandemic and of the impossibility to obtain a temporal comparison of the same cohort, we have searched the most recent literature for a historical series of HNC patients. Reference values for the EORTC QLQ-C30 module are publically available in the institutional site (https://www.eortc.org/app/uploads/sites/2/2018/02/reference_values_manual2008.pdf) but these are explicitly based on pretreatment values, i.e., they are based on a population with an active HNC. Instead, a paper published some years ago by Bjordal et al. has separately administered the QoL module to a population of 360 HNC survivors (defined as being disease-free at least from 1 year), and this was ultimately chosen as a control group [15]. Moreover, we separately evaluated the results obtained by patients who underwent total laryngectomy for laryngeal/hypopharyngeal cancer (laryngectomized population, LP) with those of other HNC survivors. This was done in order to assess if a permanent tracheostomy and its associated breathing side effects had an impact on the scores of both EORTC QLQ-C30 and the COVID-19 HNC Respiratory Questionnaire. Then, we compared the scores of the three subgroups (LP vs other HNC patients vs control group) to explore any possible differences and, finally, to explore the specific role of communication impairment, further comparison between patients with and without a voice prosthesis was undertaken in the LP subgroup.

### Statistical methods

The adequacy of the sample size was assessed by a preliminary calculation that expected deterioration in our cohort compared to historical controls. Alpha was set at 0.05, power at 0.8, and minimally important differences for the EORTC QLQ-C30 in patients with head and neck cancer were considered the anticipated differences [16]. Standard descriptive statistics were used to present data while the Shapiro-Wilk test was used to check for normality. We used unpaired two-tailed Student’s *t*-test and ANOVA to compare the mean score for each answer, while the proportion of the single value in each item was compared by chi-squared test. *p*-values of less than 0.05 were regarded as statistically significant. All statistical analyses were performed using SPSS for Apple iOS (v. 23, SPSS Inc., Armonk, NY).

## Results

One hundred and twenty-one HNC patients completed the survey, and a general description of our population and of the control group is given in Table 2. Overall, the two populations were comparable in terms of sex, history of previous head and neck radiotherapy, and cancer subsite; unfortunately, statistical comparison in terms of age could not be performed due to incomplete data in the control group.

**Table 2:** Demographics and general clinical data of our population and the control group derived from Bjordal et al. [15]. Data are expressed as absolute values and percentage in brackets. *COPD*, chronic obstructive pulmonary disease; *HNC*, head and neck cancer; *LP*, laryngectomized population; *NA*, not available; *RT*, radiotherapy

	Overall population [121]	Control group [360]	*p*-value		LP group [64]	Other HNC [57]	*p*-value
Median age (range)	67 (35–95)	61 (22–91)	-	Subgroup analysis of our population [121]	73.5 (44–95)	64 (35–87)	-
Sex			0.107				
Male	91 (75.2)	295			52	39	
Female	30 (24.8)	(81.9) 65 (18.1)			(81.2) 12 (18.8)	(68.4) 18 (31.6)	0.103
Positive smoking history	83 (68.6)	NA	-		47 (73.4)	36 (63.2)	0.224
Mean pack/years	72.4	NA	-		63.23	79.53	0.196
COPD/asthma	23 (19)	NA	-		15 (23.4)	8 (14)	0.188
Site			0.380				-
Oral cavity Oropharynx Larynx Hypopharynx Nasopharynx	34 (28.1) 12 (9.9) 63 (52) 5 (4.2) 7 (5.8)	107 (29.7) 41 (11.3) 185 (51.3) 19 (5.2) 8 (2.5)			- - 59 (92.2) 5 (7.8) -	34 (59.6) 12 (21) 4 (7.1) 0 (0) 7 (12.3)	
History of previous RT	98 (80.9)	309 (85.8)	0.201		56 (71.8)	42 (49.1)	0.053

The overall comparison between our cohort and the control group in terms of EORTC QLQ-C30 is presented in Table 3. Remarkably, the results of the physical (80.5 vs 85, *p* = 0.028), role (78 vs 84, *p* = 0.030), and emotional functioning items (76 vs 81, *p* = 0.041) were significantly different in the two groups, with worse functioning in the former. Comparing the three groups, a worse score for social (76.6 in LP vs 88.9 in HNC vs 86 in the control group, *p* = 0.006) and physical functioning (75.5 in LP vs 86.1 in HNC vs 85 in the control group, *p* = 0.001) and dyspnea (22.9 in LP vs 8.2 in HNC vs 20 in the control group, *p* = 0.008) in the LP was confirmed; the other parameters did not differ significantly (data not shown).

**Table 3:** A comparison between EORTC QLQ-C30 mean values (SD) in the cohort in the present study vs those published by Bjordal et al. and that were used as a control group [15]. *Significant difference with a *p*-value < 0.05

EORTC QLQ-C30 Scales	Overall population (121)	Control group (360)	*p*-value
Physical functioning	80.5 (21.1)	85 (18.8)	0.028*
Role functioning	78 (28.5)	84 (25.4)	0.030*
Emotional functioning	76 (24.4)	81 (22.8)	0.041*
Cognitive functioning	89 (17.3)	86 (19.8)	0.138
Social functioning	82.4 (26)	86 (22.8)	0.148
Fatigue	22.8 (26.3)	21 (23.6)	0.481
Nausea/vomiting	2.9 (10.7)	5 (13.3)	0.116
Pain	11.2 (22.3)	15 (23)	0.114
Dyspnea	16 (26.2)	20 (29.5)	0.185
Insomnia	22 (33.2)	22 (30.5)	1
Appetite loss	12.1 (24.7)	13 (25.8)	0.737
Constipation	11.4 (30.5)	11 (23.6)	0.881
Diarrhea	6.1 (17.7)	5 (16)	0.525
Financial difficulties	14.6 (29.5)	14 (27.6)	0.839
Global health status	71 (20.5)	73 (21.7)	0.374

The results of the comparison between LP and other HNC patients’ answers are reported in Table 4. In the latter group, no patient had a tracheostomy nor a percutaneous feeding tube. Regarding EORTC QLQ-C30, social (76.6 vs 88.9, *p* = 0.008) and physical functioning (75.5 vs 86.1, *p* = 0.006) were reported to be significantly worse in the LP group; dyspnea was also worse in the same group (22.9 vs 8.2 *p* = 0.003). When the COVID-19 HNC Respiratory Questionnaire items are considered, the LP reported a greater increase in mucus production (1.67 vs 1.37, *p* = 0.036) and they were more concerned that other people could be afraid of being close to them (1.67 vs 1.32, *p* = 0.020), while the other HNC patients noticed more of an improvement in air quality during the lockdown period (2.32 vs 1.89, *p* = 0.032). Asking for the positioning of facemasks in the LP group, the majority of patients put the mask on the mouth and nose (46.9%, Table 4); a third of them (35.9%) used two masks to cover also the tracheostomy; and a small percentage (7.8%) use it only on the tracheostomy. Finally, we did not find significant differences in any of the scores of both questionnaires of laryngectomized patients with and without a voice prosthesis.

**Table 4:** The comparison between LP subgroup and other HNC patients’ scores using both questionnaires. *Significant difference with a *p*-value < 0.05

	LP group (64)	Other HNC (57)	*p*-value
COVID-19 HNC Respiratory Questionnaire			
Increase in mucus production	1.67	1.37	0.036*
More cough	1.31	1.14	0.071
Improvement in air quality	1.89	2.32	0.032*
Respiratory discomfort due to the increased use of disinfectants	1.28	1.16	0.223
Warned to have a higher risk of becoming infected	2.33	2.04	0.143
Fear of others to be near	1.67	1.32	0.020*
Fear of crowded places	2.06	1.96	0.597
Where did you place the mask to go out?			
On mouth and nose On the tracheostomy On both Nowhere because I use the filter Nowhere because I thought it was unnecessary	30 (46.9%) 5 (7.8%) 23 (35.9%) 3 (4.7%) 3 (4.7%)		
EORTC QLQ-C30 Scales			
Physical functioning	75.5 (20.9)	86.1 (20.2)	0.006*
Role functioning	78.6 (25.8)	78.6 (31.5)	0.999
Emotional functioning	74.5 (22.9)	78.4 (26.2)	0.385
Cognitive functioning	88.3 (17)	89.7 (17.7)	0.639
Social functioning	76.6 (28)	88.9 (22.1)	0.008*
Fatigue	26 (23.4)	19.1 (29)	0.148
Nausea/vomiting	1.8 (6)	4.1 (14.2)	0.266
Pain	12.0 (19.6)	10.2 (25.1)	0.670
Dyspnea	22.9 (28.4)	8.2 (21.2)	0.003*
Insomnia	19.3 (30.7)	25.1 (35.8)	0.334
Appetite loss	15.1 (27.2)	8.8 (21.4)	0.155
Constipation	15.5 (34)	8.2 (23)	0.175
Diarrhea	6.8 (16)	5.3 (19.8)	0.643
Financial difficulties	10.4 (21.3)	19.3 (36.2)	0.109
Global health status	69.3 (19.1)	72.9 (22)	0.328

## Discussion

Our study, to the best of our knowledge, is the first to investigate the psychosocial impact of the current pandemic on the HNC survivors. Besides the risk of being infected, the impact of COVID-19 on the delivery of cancer care has apparently exacerbated the sense of frailty, isolation, and consequent deterioration in the QoL of these patients. At a time when surgeries, chemotherapy sessions, and follow-up visits are being postponed because of the disrupted healthcare systems, cancer patients must be reasonably considered a population at risk of significant distress [17–19]. This psychological burden associated with the direct and indirect effects of COVID-19 must not be overlooked because, in a recent systematic review, depression seemed to be an independent predictor of survival in cancer patient cohort [20]. A large amount of (sometimes) conflicting information about the risk for cancer patients of being infected, and of developing severe COVID-19, come from both the mass media and the published literature: no wonder that this has reasonably generated only more confusion and fear [21–24].

Our results show a deterioration in both physical and emotional functioning during the lockdown and, more importantly, the differences in the respective scales can be considered clinically significant [16]. This might be explained because government restrictions have forced the entire population to reduce their activities and to spend entire weeks being locked-up at home. This had a negative impact on both psychological and physical states, as it has been already reported in numerous studies on non-cancer populations [25–27]. In addition, a large proportion of our cohort was old and elderly subjects seem particularly vulnerable in this respect. Actually, some authors have expressed concerns about the level of independence of this subpopulation after the end of quarantine, given no appropriate campaign to promote physical activity has ever been promoted [27]. Some specific results of our study are also intriguing: for instance, we would have expected a profound decline in the social functioning scale. A possible explanation might be linked to the perception that social contacts have completely been abolished in the lockdown period for everybody; thus, our cohort might have underestimated their specific condition when reported to the global situation.

Preliminary data on the effect of the current pandemic on people with active cancer are becoming available. A study from Poland has recently analyzed 238 patients with stage III/IV of different types of cancer undergoing chemotherapy by using EORTC QLQ-C30: compared to reference values, the global QoL, and the cognitive and social functioning were significantly lower during the COVID-19 pandemic, as well as insomnia, fatigue, and loss of appetite items appeared to be worse [11]. Similarly, other studies analyzed the psychological status of COVID-19 in specific cancer subpopulations (gynecological and hematological tumors), confirming a deterioration in terms of QoL and an increase of anxiety and distress symptoms [28–31]. Such results may instead not apply to all fields of head and neck oncology: for instance, Falcone et al. did not find any significant deterioration in EORTC QLQ-C30 scores of patients with thyroid malignancies during the COVID-19 pandemic [32]. They also found no intraindividual significant changes in terms of global health and of functional statuses [32]. Such negative findings might be explained by the fact that thyroid cancer is notably an indolent tumor, and therefore, these patients could feel less at risk of contagion compared to the population with squamous cell HNC.

We must acknowledge, though, that all the aforementioned reported differences in several EORTC scales might not be enough to investigate the effects of COVID-19 on HNC survivors. As it was elegantly pointed out some years ago, these questionnaires may not fully depict the many complex factors (cultural background, personal expectations, etc.) which can influence the subjective answers to these statistical tools [33]. Nonetheless, our and others’ findings highlight the importance of focusing on the psychological health of patients with cancer during this unprecedented pandemic. As reported by Wang et al., despite a high prevalence of mental health problems, only a small percentage of cancer patients did seek help for psycho-oncological counseling during the first months of the last year [12].

Considering the laryngectomized population only, it was shown some years ago that these patients show a significant deterioration of almost all the EORTC QLQ-C30 domains immediately after the surgery, and without returning to baseline over time [34]. In the COVID-19 era, the fear and concerns related to the risk of being infected and the lockdown itself might have actually worsened this situation. The low scores in the social functioning for the LP group may in fact reflect the increased difficulties in family and social life during these months. Furthermore, these patients reported a greater perception that others were afraid of being close to them and we can hypothesize that tracheostomy and the frequent coughing attacks have alerted and scared people around them. The capacity of cough to disperse potentially infectious viral particles, through respiratory droplets, is well-known by the general population. And it is one of the key elements that can give rise to a true social stigma [35, 36].

Regarding patients with vocal prosthesis, they are known to require demanding clinical management, even though it was demonstrated how they can largely benefit from tele-visits to solve minor troubles and without coming to the office [37]. In the present paper, no differences emerged from the comparison between LP with and without a voice prosthesis, highlighting how a deficit in communication may have a lower impact on QoL compared to the feeling of being at risk of infection because of tracheostomy. Moreover, although all had been instructed about the importance of protecting their stoma, many of the patients have reported wearing an additional mask over their mouth and nose, only for not being reprimanded by others. Finally, the improvement in air quality in Italy was a well-known positive effect of the lockdown, thanks to the closure of industrial and human activities [38, 39]. How this unique situation is related to the reported differences between LP and other HNC in terms of respiratory comfort should be clarified by future and specifically addressed studies [40].

The present work has some limitations: first, the comparison with a historical population has always some intrinsic flaws because of the heterogeneity of the two groups and of the fact that not all confounders could be controlled; secondly, because this is an anonymous questionnaire compiled by patients, we were not able to retrieve precise data regarding the tumor staging and the type of surgery performed. Third, the respiratory questions were not formally validated and they do suffer from the well-known psychometric limitations of this kind of tool. Finally, our survey was conducted when no clinics and structural support for HNC patients were available to help them to cope with an unprecedented situation. Now that healthcare systems have adapted to the ongoing pandemic, future research investigating how the situation changes over time in this group will be of great interest.

## Conclusions

HNC survivors, and in particular those who underwent a total laryngectomy, appear to represent a population most at risk for a deterioration in the QoL because of the COVID-19 pandemic and independently of the fact of being infected. As our task is to take care not only of the physical but also of the mental health of our patients, we strongly advise clinicians to evaluate how HNC survivors are living these unprecedented times, in order to promptly identify psychological distress and to favor all types of support.

## Data Availability

Data are available upon reasonable request to the corresponding author.

## Code availability

N/A.
